# LINC00470 promotes tumour proliferation and invasion, and attenuates chemosensitivity through the LINC00470/miR‐134/Myc/ABCC1 axis in glioma

**DOI:** 10.1111/jcmm.15846

**Published:** 2020-09-11

**Authors:** Changwu Wu, Jun Su, Wenyong Long, Chaoying Qin, Xiangyu Wang, Kai Xiao, Yang Li, Qun Xiao, Junquan Wang, Yimin Pan, Qing Liu

**Affiliations:** ^1^ Department of Neurosurgery Xiangya Hospital Central‐South University Changsha China; ^2^ Institute of Anatomy University of Leipzig Leipzig Germany; ^3^ Institute of Skull Base Surgery and Neuro‐oncology at Hunan Changsha China

**Keywords:** ABCC1, ceRNA, glioma, LINC00470, miR‐134, MYC, temozolomide sensitivity

## Abstract

Glioma is the most common primary malignant tumour in the brain; temozolomide (TMZ) is the most prevalent chemotherapeutic drug currently used to combat this cancer. We reported previously that the long intergenic non‐protein coding RNA 470 (LINC00470) is a novel prognostic biomarker for glioma and promotes the tumour growth in an intracranial transplantation mouse model. However, the effects of LINC00470 on glioma cell proliferation, invasion and TMZ chemosensitivity, as well as its molecular mechanism, remain unclear. In this study, we found elevated expression levels of LINC00470 and MYC in glioma tissues and cells and decreased expression of microRNA‐134 (miR‐134). Functional studies have shown that LINC00470 promotes proliferation and invasion, and attenuates chemosensitivity of glioma cells, while miR‐134 exerts the opposite effect. In the rescue experiments, the tumorigenic effect of LINC00470 was offset by miR‐134. In the mechanism study, we found that LINC00470 was a competitive endogenous RNA (ceRNA) of miR‐134 and that miR‐134 can directly target MYC and negatively regulate its expression. Furthermore, MYC was positively correlated with ATP‐binding cassette subfamily C member 1 (ABCC1) expression in glioma cells and MYC up‐regulated ABCC1 expression. Further studies found that LINC00470 regulated MYC by sponging miR‐134 to regulate the expression of ABCC1. We concluded that LINC00470 promoted the expression of MYC and ABCC1 by suppressing miR‐134, thus promoting glioma cell proliferation and invasion, and attenuating TMZ chemosensitivity. Moreover, the LINC00470/miR‐134/MYC/ABCC1 axis constitutes a potential therapeutic target.

## 1 INTRODUCTION

Glioma is the most common primary malignant tumour of the human central nervous system, accounting for more than half of the primary malignant tumours in the brain.^1^ Low‐grade gliomas have a better prognosis when surgery is combined with radiotherapy and chemotherapy; however, the 5‐year survival rate remains less than 5% in high‐grade gliomas such as glioblastoma multiforme (GBM). This is closely related to the highly invasive and rapid proliferation of GBM.^2^ Therefore, it is particularly important to further explore the molecular mechanism of the malignant progression of glioma. Temozolomide (TMZ) is a DNA‐alkylating agent that crosses the blood‐brain barrier and is considered a first‐line treatment for GBM.^3^ Although TMZ has been shown to suppress tumour growth and prolong the survival of GBM patients, resistance to it is common, causing many treatment failures.^4^, ^5^ Multidrug resistance (MDR) is the main reason for this chemotherapy resistance.^6^

Long non‐coding RNAs (lncRNAs), comprising a set of non‐coding RNAs greater than 200 nucleotides in length, have been shown to play an important role in the occurrence and development of a variety of tumours, including gliomas.^7^, ^8^ Long intergenic non‐protein coding RNA 470 (LINC00470) is a lncRNA located on chromosome 18p11.32.^9^ In our previous studies, we found that LINC00470 activated the AKT signalling pathway and promoted the tumour growth of intracranial transplantation mouse model with glioma.^10^ However, the role and mechanism of LINC00470 in glioma must be further elucidated; in particular, it remains unclear whether LINC00470 affects the chemosensitivity of glioma.

Unlike lncRNAs, microRNAs (miRNAs) contain only 22 nucleotides and regulate gene expression post‐transcriptionally by targeting the 3' untranslated region (3'UTR) of messenger RNA (mRNA).^11^ miRNAs regulate the expression of approximately 30%‐50% of human genes.^12^, ^13^ In addition, more than 50% of miRNAs are involved in the occurrence and development of human tumours by directly acting on oncogenes or tumour suppressor genes.^12^ Recent studies have shown that microRNA‐134 (miR‐134) plays an important role in tumour cell proliferation, apoptosis, invasion and metastasis. For example, miR‐134 inhibits the proliferation of lung cancer cells by down‐regulating the expression of *EGFR*.^14^ It is known that miR‐134 is down‐regulated in gliomas and is negatively correlated with World Health Organization (WHO) glioma grades; the mechanism may involve the promotion of glioma cell migration and invasion by targeting the *KRAS* gene and activating the ERK signalling pathway.^15^, ^16^ However, a deeper understanding of the miR‐134 mechanism in glioma remains to be investigated.

Recent studies report that lncRNA can act as a special 'sponge' to adsorb miRNA, thereby reducing the regulatory effect of miRNA on mRNA.^17^ In our present study, we confirmed the role of LINC00470 as an oncogene in gliomas and found that LINC00470 acts as competing endogenous RNA (ceRNA) to inhibit miR‐134 to further regulate the expression of the oncogene *MYC*. We also found a positive correlation between the expression of MYC and the expression of ATP‐binding cassette subfamily C member 1 (ABCC1, also known as multidrug resistance protein 1) in gliomas and that LINC00470 affected the chemosensitivity of glioma cells by regulating the expression of ABCC1 by interacting with miR‐134. Our results provide a new understanding of the role and mechanism of the LINC00470/miR‐134/MYC/ABCC1 axis in glioma, which may contribute to the development of novel therapeutic targets and improve the chemosensitivity of glioma.

## 2 MATERIALS AND METHODS

### 2.1 Public database, tissue samples and cell lines

The expression levels of MYC and ABCC1 in 541 GBMs were obtained using a gene expression array (Affymetrix U133A; Affymetrix/Thermo Fisher Scientific, Santa Clara, CA, USA) referenced in The Cancer Genome Atlas (TCGA) database (https://portal.gdc.cancer.gov/exploration). Thirty‐two glioma tissues and seven normal brain tissues were collected at the Department of Neurosurgery, Xiangya Hospital, Central South University, Hunan, China. After surgical resection, fresh specimens were sent to the Department of Pathology for neuropathological evaluation and the remaining specimens were frozen in liquid nitrogen immediately. The glioma group comprised eight WHO II, eight WHO III and sixteen WHO IV tumours and the normal brain tissues were taken from patients with brain trauma. Clinical parameters of thirty‐two glioma patients are listed in Table S1. This study was approved by the Ethics Committee of Xiangya Hospital, Central South University, Hunan, China, and written informed consent was provided by all of the patients.

Astrocytoma cell lines U251 and U87, human normal glial cell line HEB, and human renal epithelial cell line 293T were purchased from the Chinese Academy of Sciences Cell Bank (Shanghai, China), and the cell lines were all subjected to short tandem repeat profiling. U251, HEB and 293T cell lines were cultured in DMEM high glucose medium (HyClone, Logan, UT, USA) containing 10% FBS (Gibco, Gaithersburg, MD, USA). The U87 cell line was cultured in MEM (HyClone) containing 10% FBS. All cells were incubated in a humidified incubator at 37°C, 5% CO_2_.

### 2.2 miRNA mimics, DNA plasmid and transfection

miR‐134 mimics and miR‐NC were chemically synthesized by GenePharma (Shanghai, China). Overexpression plasmids LINC00470, MYC and ABCC1 and control plasmid vector were synthesized by GeneChem (Shanghai, China). The atlas of the luciferase reporter plasmid was provided in Figure S1. Cell suspensions were prepared using pancreatin‐digested U87 and U251 cells and complete medium. Then, the cells were seeded in 6‐well plates and incubated for 24h. Before transfection, the cells were washed with PBS solution and incubated with fresh culture medium without serum and antibiotics. Cell transfection was performed with Lipofectamine® 3000 (Invitrogen/Thermo Fisher Scientific, Carlsbad, CA, USA) per the manufacturer's instructions.

### 2.3 Quantitative reverse transcription‐PCR (RT‐qPCR) and Western blotting

RT‐qPCR and Western blotting were performed as described previously.^18^ The following RT‐qPCR primers were used: GAPDH, forward 5′‐AATGGGCAGCCGTTAGGAAA‐3′, reverse 5′‐GCGCCCAATACGACCAAATC‐3′; U6, forward 5′‐CTCGCTTCGGCAGCACA‐3′, reverse 5′‐AACGCTTCACGAATTTGCGT‐3′; LINC00470, forward 5′‐CGTAAGGTGACGAGGAGCTG‐3′, reverse 5′‐GGGGAATGGCTTTTGGGTCA‐3′; miR‐134, forward 5′‐ATTGATACCTGTGGGCCACCTA‐3′, reverse 5′‐CACCAGCAGCGACTCTGA‐3′; MYC, forward 5′‐CCCTCCACTCGGAAGGACTA‐3′, reverse 5′‐GCTGGTGCATTTTCGGTTGT‐3′; and ABCC1, forward 5′‐AACCTGGACCCATTCAGCC‐3′, reverse 5′‐GACTGGATGAGGTCGTCCGT‐3′. The expression levels of LINC00470, MYC, ABCC1 and miR‐134 were calculated by 2^−ΔΔ^ *^CT^* method. The following antibodies were used for Western blot analysis: mouse monoclonal antibody to GAPDH (MilliporeSigma, Burlington, MA, USA), rabbit polyclonal antibody to MYC (Sangon Biotech, Shanghai, China) and mouse monoclonal antibody to ABCC1 (Santa Cruz Biotechnology, Santa Cruz, CA, USA).

### 2.4 Cell viability assay

Cell activity was measured with a CCK8 assay. The transfected cells were cultured for 24 hours, then seeded in 96‐well plates (2000 cells per well). After the cells attached, they were cultured for different time periods (0, 1, 2 and 3 days) after different treatments (with or without TMZ). Ten microlitres of CCK8 reagent was added to each well, and the absorbance was measured at 450 nm after incubation at 37°C for 3 hours.

### 2.5 Cell invasion assay

Cell invasion was measured by Matrigel® Transwell® chambers (Corning Inc, Corning, NY, USA); the transfected U87 and U251 cells were digested with pancreatin to prepare cell suspensions. 2 × 10^4^ cells were seeded in the upper chamber and incubated in 5% serum medium and the lower chamber in 15% serum medium. After incubation for 24 to 48 hours, the Matrigel® in the upper chamber was wiped off with a cotton swab, while cells on the lower side were observed using a microscope. Then, the cells on the chamber membrane were fixed with 4% paraformaldehyde and stained with 10% crystal violet. Five visual fields were randomly counted in each chamber.

### 2.6 Immunohistochemistry

Immunohistochemistry was carried out as described previously.^19^ The following antibody was used: rabbit polyclonal antibody to MYC (Sangon Biotech).

### 2.7 Dual‐luciferase reporter assay

293T cells were plated in a 12‐well plate and then co‐transfected with LINC00470‐Wt/MYC‐Wt or LINC00470‐Mut/MYC‐Mut and miR‐134 mimics or miR‐134 negative control (NC). After transfection, cells were incubated for 24 hours. The cells were collected and analysed using the Dual‐Luciferase Reporter Assay System (Promega, Madison, WI, USA). The luciferase activity was calculated as the ratio of firefly luciferase intensity/Renilla luciferase intensity.

### 2.8 Statistical analysis

All experiments were repeated three times, and data were statistically analysed using GraphPad Prism 5 (La Jolla, CA, USA) and SPSS version 20.0 (IBM Corp., Armonk, NY, USA). Descriptive statistical analysis was used to ensure normality. The Levene test was used to test the homogeneity of variance. Differences between the different groups were assessed using Student's t test or one‐way ANOVA. When the variance was unequal, t' test or Welch's ANOVA was used and Kruskal‐Wallis test was used for verification. The correlation between the expression levels of the two genes was analysed using Pearson's chi‐squared test. A *P*‐value of <0.05 was considered to be statistically significant.

## 3 RESULTS

### 3.1 LINC00470 was up‐regulated in glioma tissues and cell lines

**Figure 1:**
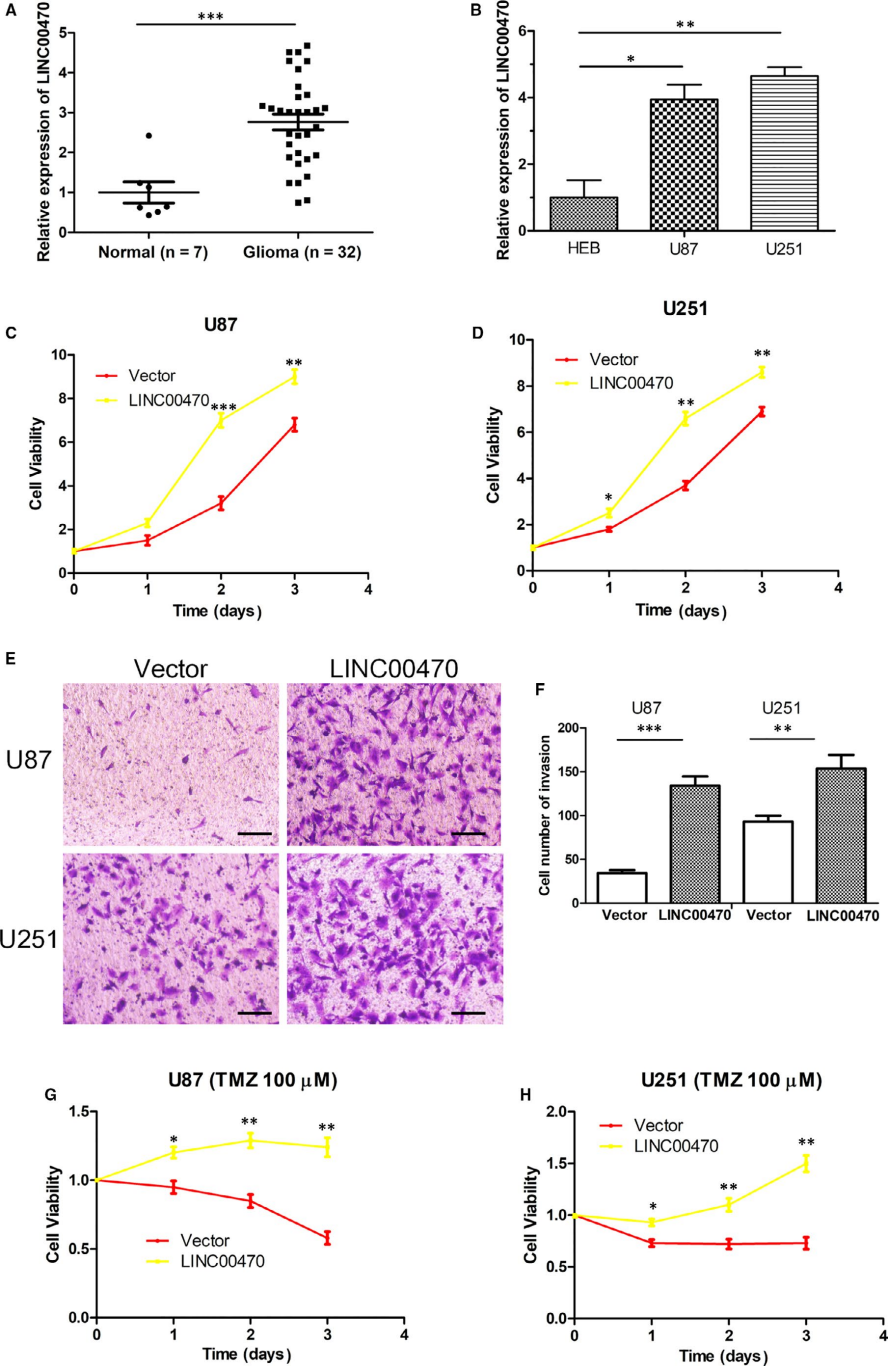
RT‐qPCR showed that LINC00470 was up‐regulated in glioma and promoted glioma cell proliferation, invasion and TMZ resistance. A, The expression of LINC00470 in glioma tissues (n = 32) was significantly up‐regulated relative to normal brain tissues (n = 7). GAPDH was the internal control. B, The relative expression of LINC00470 in glioma cell lines U87 and U251 was higher than that in normal cell line HEB. GAPDH was the internal control. C,D, The cell viability was increased when LINC00470 was overexpressed in U87 and U251 cells compared with controls. E,F, Overexpression of LINC00470 enhanced the invasive ability of U87 and U251 cells. The photographs were taken at 200 × magnification. Scale bar: 100 μm. G,H, LINC00470 enhanced the chemoresistance of U87 and U251 cells to TMZ. The data shown represent the mean ± SD of at least three independent experiments. *P < 0.05; **P < 0.01; ***P < 0.001. TMZ, temozolomide; RT‐qPCR, quantitative reverse transcription

In this study, we analysed the expression of LINC00470 in 32 glioma tissues and 7 normal brain tissues from the Department of Neurosurgery, Xiangya Hospital, China, by RT‐qPCR. We found that the expression of LINC00470 in glioma tissues was significantly up‐regulated relative to normal brain tissues (Figure 1A). Meanwhile, compared with normal brain tissue cell line HEB, the expression levels of LINC00470 in glioma cell lines U87 and U251 were also up‐regulated (Figure 1B). These results indicated that LINC00470 may play a biological role in the development and/or progression of glioma.

### 3.2 LINC00470 promoted glioma cell proliferation and invasion, and attenuated TMZ chemosensitivity

In order to explore the biological role of LINC00470 in glioma cells, an LINC00470 overexpression plasmid was transfected into cell lines U87 and U251. A CCK8 assay showed that the cell viability was significantly increased when LINC00470 was overexpressed in U87 and U251 cells compared with controls (Figure 1C‐D). Meanwhile, a Transwell® invasion assay indicated that overexpression of LINC00470 significantly enhanced the invasive ability of glioma cells (Figure 1E‐F). We treated U87 and U251 cells with 100 μmol/L TMZ prior to the CCK8 assay and found that overexpression of LINC00470 attenuated the inhibitory effect of TMZ on glioma cell viability. In other words, LINC00470 attenuated chemosensitivity of glioma cells to TMZ (Figure 1G‐H). These results suggest that LINC00470 can be considered an oncogene that promotes the proliferation and invasion, and attenuates TMZ chemosensitivity of glioma cells.

### 3.3 LINC00470 was a sponge of miR‐134

**Figure 2:**
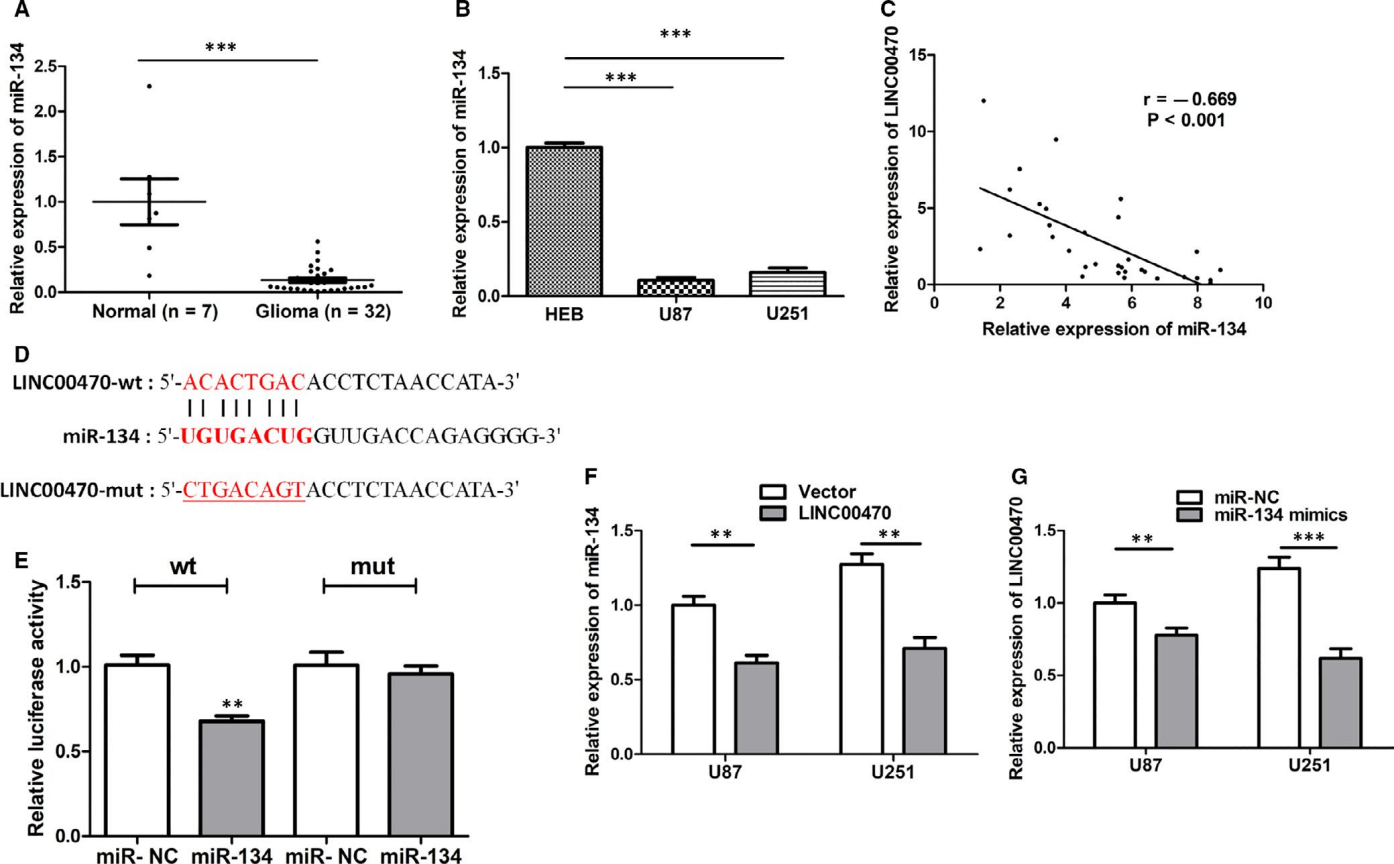
LINC00470 was a sponge of miR‐134. A,B, The relative expression of miR‐134 was down‐regulated in glioma tissues and cell lines. U6 was the internal control. C, Pearson's chi‐squared test showed a significant negative correlation between the expression of LINC00470 and miR‐134 in glioma tissues. D, miR‐134 was predicted to be a target miRNA of LINC00470. E, The luciferase activity of 239T cells co‐transfected with miR‐134 mimics and LINC00470‐Wt was significantly reduced, while the luciferase activity of 239T cells co‐transfected with miR‐134 mimics and LINC00470‐Mut was scantly affected. F, The expression of miR‐134 was down‐regulated when we overexpressed LINC00470 in U87 and U251 cells. U6 was the internal control. G, The expression of LINC00470 was down‐regulated when we overexpressed miR‐134. GAPDH was the internal control. The data shown represent the mean ± SD of at least three independent experiments.*P < 0.05; **P < 0.01; ***P < 0.001. NC, negative control

RT‐qPCR analysis showed that the relative expression of miR‐134 was significantly down‐regulated in glioma tissues, while the expression of miR‐134 in U87 and U251 cells was also down‐regulated relative to HEB cells (Figure 2A‐B). In addition, Pearson's chi‐squared test revealed a significant negative correlation between the expression levels of LINC00470 and miR‐134 in thirty‐two glioma tissues (Figure 2C). Previous studies report that lncRNAs interact with miRNAs as sponges to regulate their biological functions.^17^ To determine whether LINC00470 and miR‐134 also exhibit similar interactions in gliomas, we first predicted the binding site of LINC00470 and miR‐134 using bioinformatics analysis (Figure 2D). Furthermore, in the dual‐luciferase reporter assay, we found that the luciferase activity of 239T cells co‐transfected with miR‐134 mimics and LINC00470‐Wt was significantly reduced, while the luciferase activity of 239T cells co‐transfected with miR‐134 mimics and LINC00470‐Mut was scantly affected (Figure 2E). In addition, expression of miR‐134 was down‐regulated when we overexpressed LINC00470 in glioma cells (Figure 2F), whereas expression of LINC00470 was down‐regulated when we overexpressed miR‐134 (Figure 2G). These data indicated that miR‐134 is a direct target miRNA of LINC00470 and confirmed the mutual regulatory relationship between LINC00470 and miR‐134.

### 3.4 miR‐134 suppressed glioma cell proliferation and invasion and enhanced TMZ sensitivity

**Figure 3:**
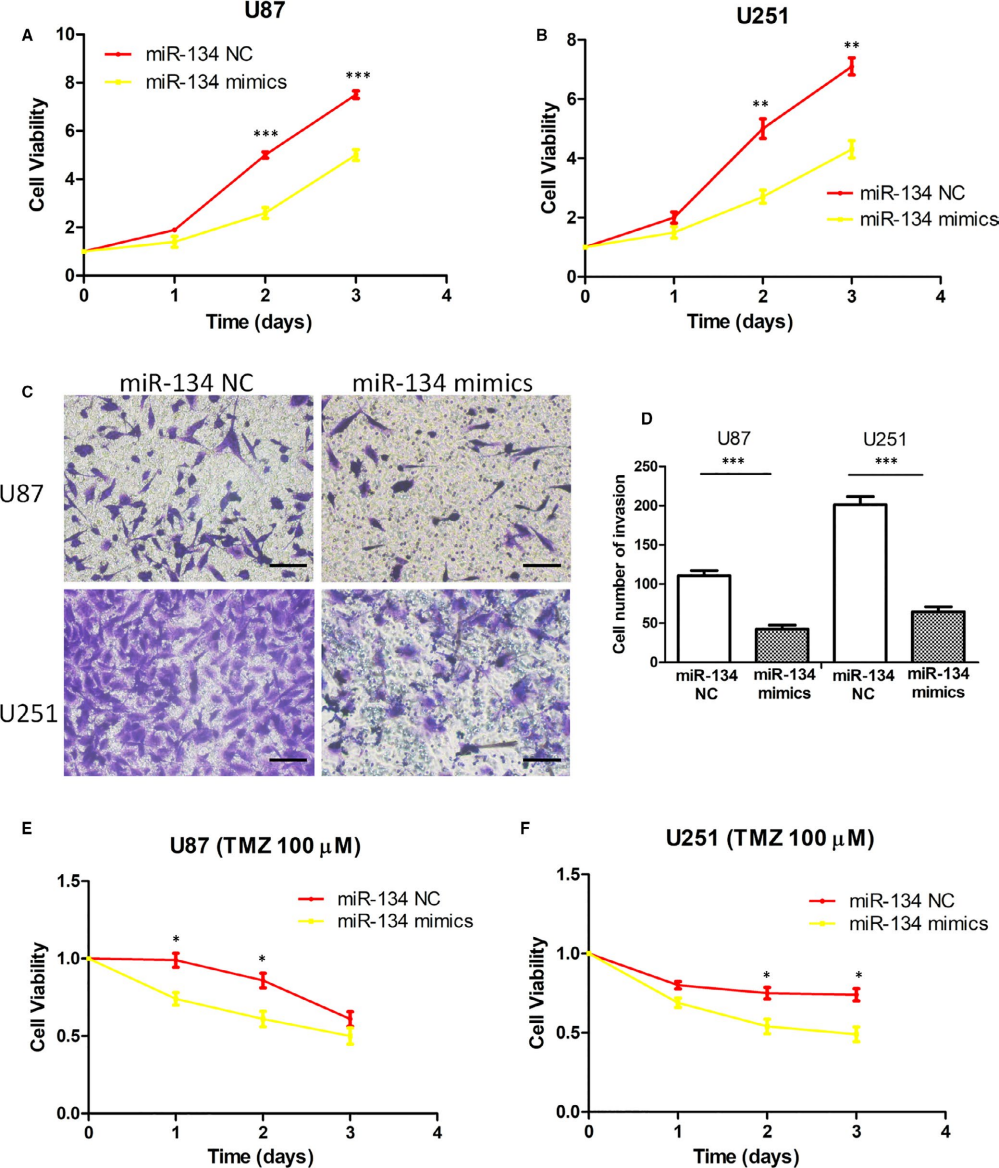
miR‐134 suppressed glioma cell proliferation and invasion and enhanced TMZ sensitivity. A,B, The cell viability was decreased when miR‐134 was overexpressed in U87 and U251 cells compared with controls. C,D, miR‐134 mimics decreased U87 and U251 cell invasion ability. The photographs were taken at 200 × magnification. Scale bar: 100 μm. E,F, Overexpressed miR‐134 enhanced the sensitivity of U87 and U251 cells to TMZ. The data shown represent the mean ± SD of at least three independent experiments. *P < 0.05; **P < 0.01; ***P < 0.001. TMZ, temozolomide; NC, negative control

From our previous results (Figure 2A‐B), we determined that miR‐134 was down‐regulated in glioma tissues and cell lines. Furthermore, we confirmed that miR‐134 mimics induced decreased glioma cell activity and invasion ability using CCK8 and Transwell^®^ assays (Figure 3A‐D). At the same time, we also found that overexpressed miR‐134 enhanced the sensitivity of glioma cells to TMZ (Figure 3E‐F). These data confirmed that miR‐134 is a tumour suppressor in glioma cell lines and affects the chemosensitivity of glioma cells.

### 3.5 miR‐134 mediated the tumour‐promoting effect of LINC00470 in glioma cells

**Figure 4:**
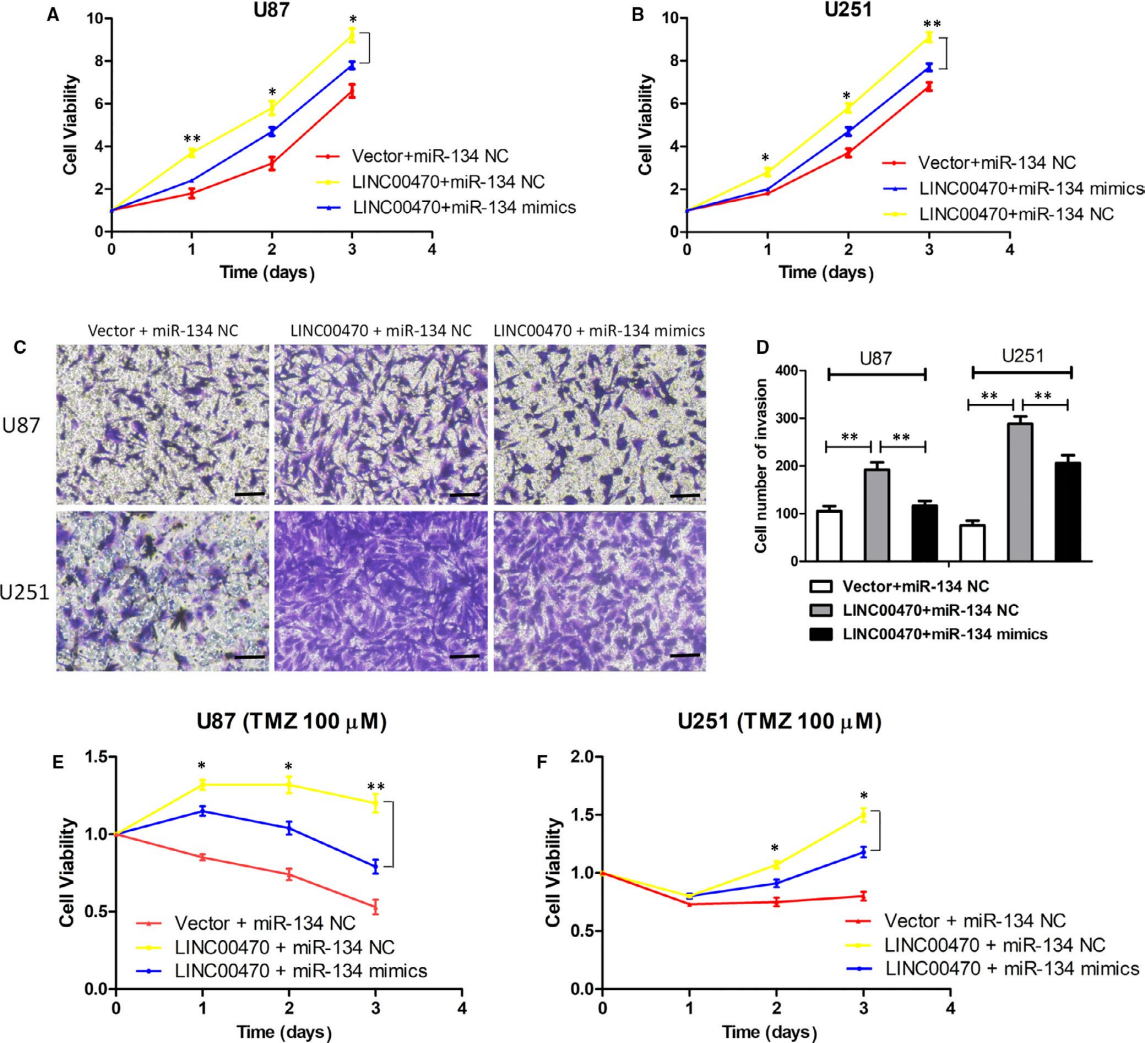
miR‐134 mediated the tumour‐promoting effect of LINC00470 in glioma cells. A,B, Rescue experiments showed that cell proliferation induced by overexpression of LINC00470 was attenuated by miR‐134 mimics. C,D, The cell invasion ability promoted by LINC00470 overexpression was rescued by miR‐134 mimics. The photographs were taken at 200 × magnification. Scale bar: 100 μm. E,F, The enhancement of TMZ resistance induced by LINC00470 overexpression was counteracted by miR‐134 mimics. The data shown represent the mean ± SD of at least three independent experiments. *P < 0.05; **P < 0.01. TMZ, temozolomide; NC, negative control

To clarify whether the tumour‐promoting effect of LINC00470 in glioma cells can be mediated by miR‐134, we performed rescue experiments. We transfected miR‐134 mimics into LINC00470‐overexpressing cells and found that cell proliferation induced by overexpression of LINC00470 was attenuated by miR‐134 mimics (Figure 4A‐B). Unsurprisingly, as shown in Figure 4C‐D, miR‐134 mimics rescued the cell invasion ability promoted by LINC00470 overexpression. At the same time, we also found that the reduction of TMZ chemosensitivity induced by LINC00470 overexpression was counteracted by miR‐134 mimics (Figure 4E‐F). The above data indicated that the tumour‐promoting effects of LINC00470 on glioma were mediated by miR‐134 and that miR‐134 overexpression largely reversed glioma cell proliferation and invasion, and reduction of TMZ chemosensitivity promoted by LINC00470.

### 3.6 Overexpression of LINC00470 up‐regulated the expression of MYC by regulating miR‐134

**Figure 5:**
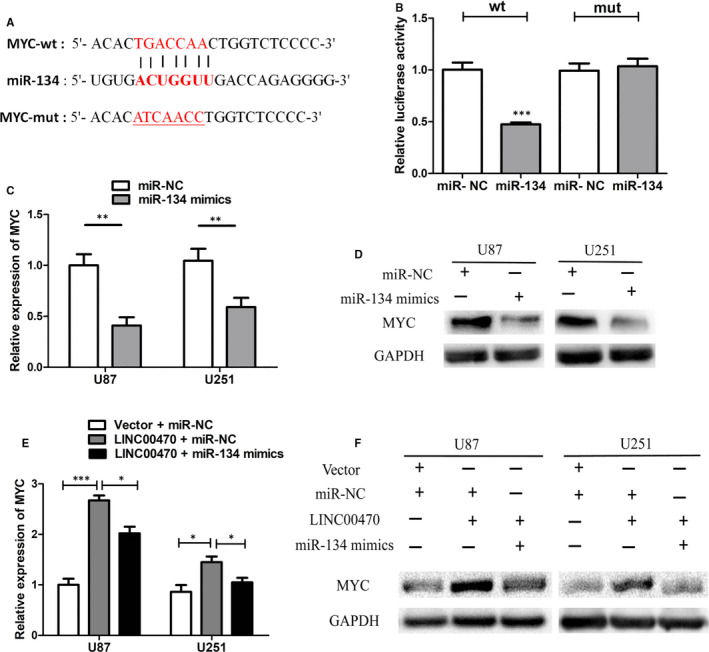
Overexpression of LINC00470 up‐regulated the expression of MYC by regulating miR‐134. A, MYC was predicted to be a direct target of miR‐134. B, The luciferase activity of miR‐134 mimics and MYC‐Wt co‐transfected cells was inhibited, while the luciferase activity of miR‐134 mimics and MYC‐Mut co‐transfected cells remained unchanged. C,D, miR‐134 mimics reduced the expression of MYC at both the mRNA and protein levels in U87 and U251 cells. GAPDH was the internal control. E,F, LINC00470 overexpression up‐regulated the expression of MYC at both the mRNA and protein levels in U87 and U251 cells. When miR‐134 mimics were transfected into LINC00470‐overexpressing cells, the up‐regulation of MYC expression was offset. GAPDH was the internal control. The data shown represent the mean ± SD of at least three independent experiments. *P < 0.05; **P < 0.01; ***P < 0.001. NC, negative control

**Figure 6:**
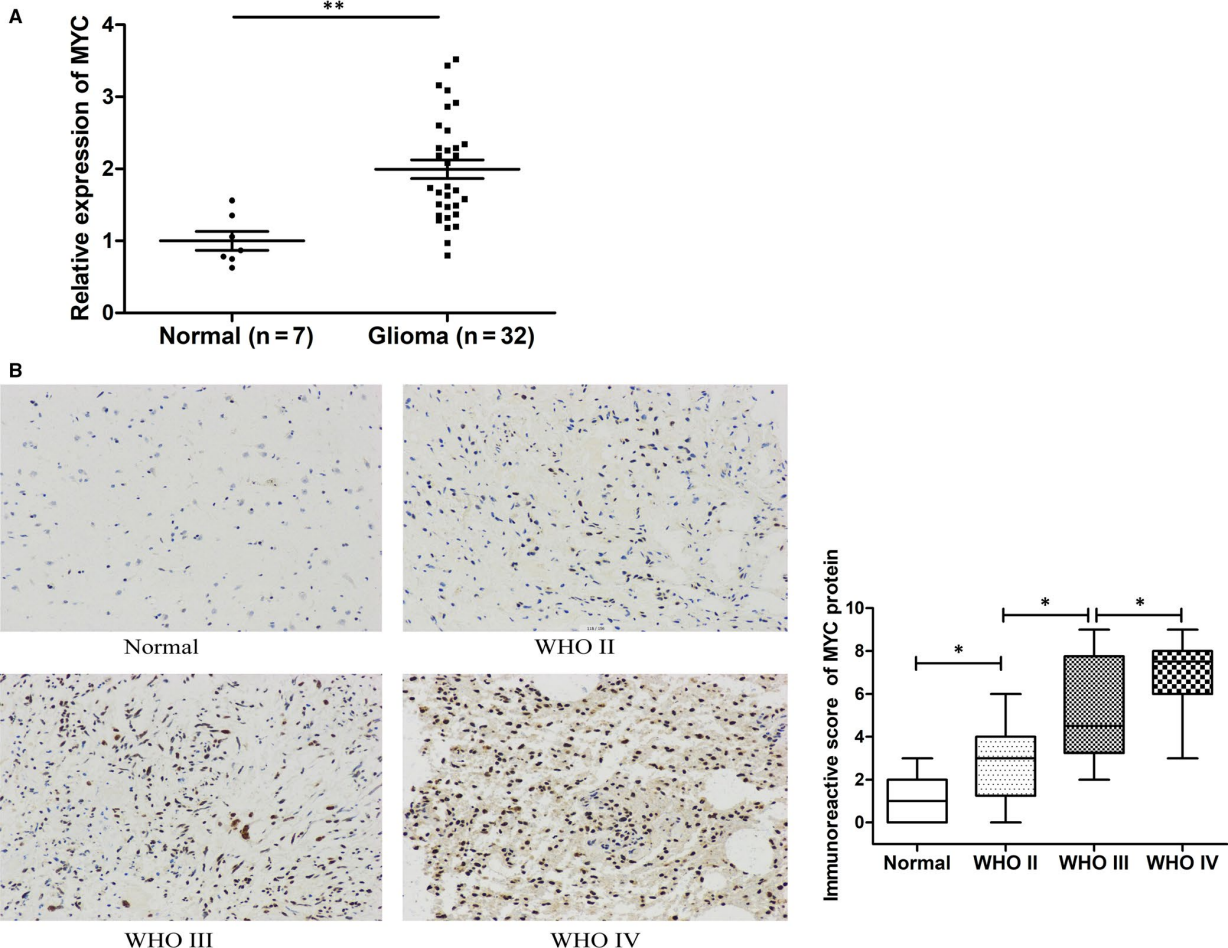
MYC was up‐regulated in glioma tissues. A, The mRNA expression level of MYC was elevated in 32 glioma tissues. GAPDH was the internal control. B, Immunohistochemical staining images of grade II, III and IV glioma tissues and non‐tumour brain tissues (magnification × 200). The immunohistochemical score indicated that MYC protein expression was dependent on WHO grade. Data are represented as mean ± SEM.*P < 0.05; **P < 0.01

It is well known that *MYC*, as a proto‐oncogene, exhibits elevated levels of expression in most human tumours—including glioma—and promotes glioma growth.^20^ Through bioinformatics analysis, we found that a binding site exists between miR‐134 and the MYC 3’UTR (Figure 5A). To confirm that MYC was a direct target of miR‐134, we performed a dual‐luciferase reporter assay. As shown in Figure 5B, the luciferase activity of miR‐134 mimics and MYC‐Wt co‐transfected cells was inhibited, while the luciferase activity of miR‐134 mimics and MYC‐Mut co‐transfected cells remained unchanged, which verified that MYC was the direct target of miR‐134. Furthermore, as expected, the transfection of miR‐134 mimics reduced the expression of MYC at both the mRNA and protein levels in glioma cells (Figure 5C‐D). In order to clarify whether MYC was involved in the tumour‐promoting role of LINC00470, we analysed the expression level of MYC in LINC00470‐overexpressing glioma cells. As shown in Figure 5E‐F, both the MYC mRNA and protein expression levels were up‐regulated by LINC00470 overexpression. Meanwhile, when miR‐134 mimics were transfected into LINC00470‐overexpressing cells, the up‐regulation of MYC expression was offset (Figure 5E‐F). These results suggested that LINC00470 up‐regulated the expression of MYC by regulating miR‐134. Besides, in our study, we also confirmed that the mRNA expression level of MYC was elevated in glioma tissues (Figure 6A). Meanwhile, we confirmed that the protein expression of MYC in glioma was significantly up‐regulated and positively correlated with pathological grade (Figure 6B).

### 3.7 Possible mechanisms of LINC00470 and miR‐134 affecting glioma cell TMZ chemosensitivity

**Figure 7:**
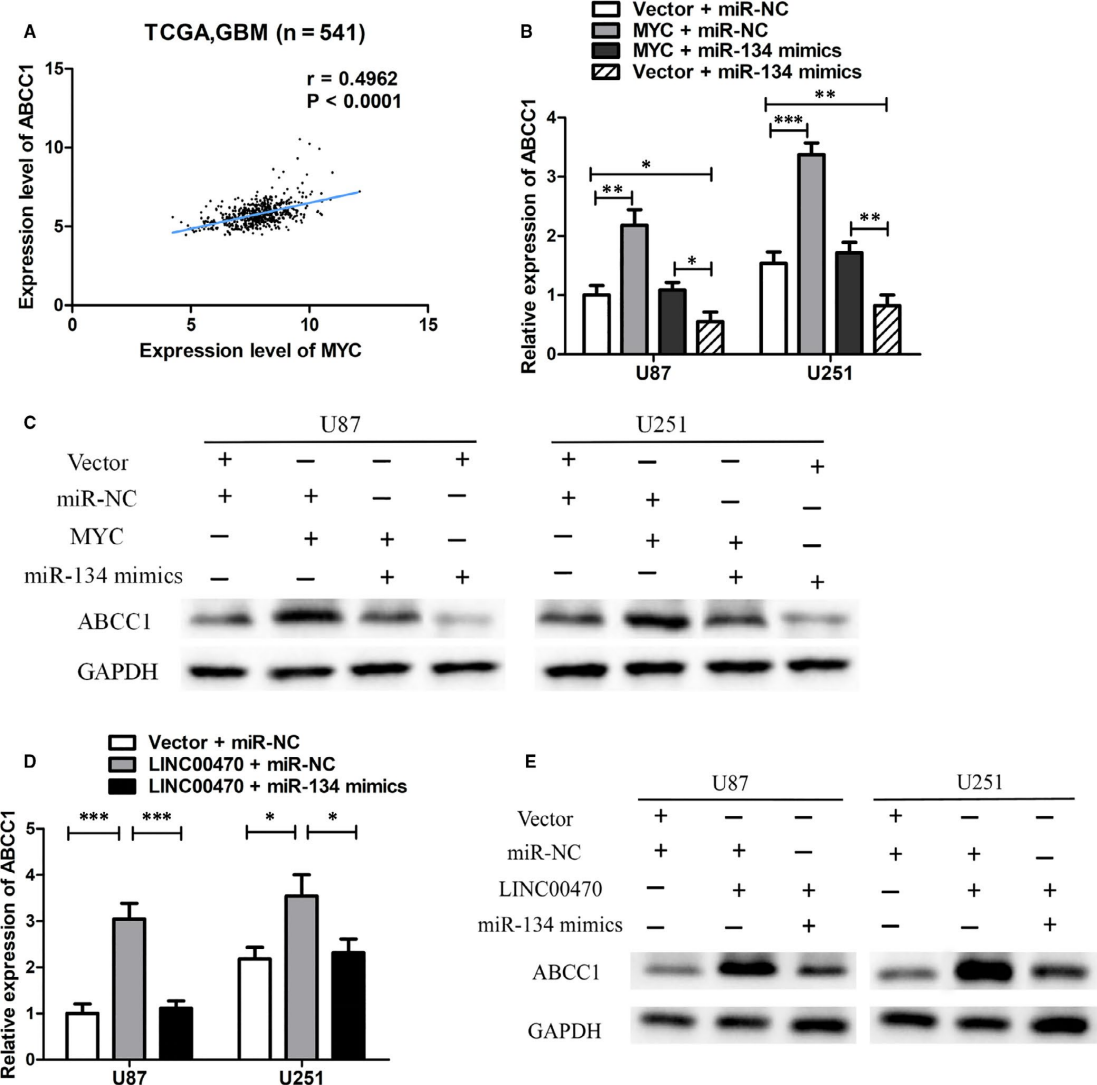
Possible mechanisms of LINC00470 and miR‐134 affecting glioma cell TMZ resistance. A, A significant positive correlation between MYC expression and multidrug resistance gene ABCC1 expression in 541 GBM samples was found by using TCGA database. B,C, Overexpression of MYC increased the expression of ABCC1 at both the mRNA and protein levels; on the contrary, overexpression of miR‐134 reduced the expression of ABCC1. The down‐regulation of ABCC1 induced by miR‐134 was rescued when we co‐transfected miR‐134 mimics and MYC plasmid into U87 and U251 cells. GAPDH was the internal control. D,E, The expression of ABCC1 at both the mRNA and protein levels was up‐regulated in LINC00470‐overexpressing cells and that this up‐regulation was counteracted when we transfected miR‐134 mimics into LINC00470‐overexpressing cells. GAPDH was the internal control. The data shown represent the mean ± SD of at least three independent experiments. *P < 0.05; **P < 0.01; ***P < 0.001. NC, negative control; GBM, glioblastoma multiforme

The results reported above have shown that LINC00470 regulated the expression of the oncogene *MYC* through miR‐134, thereby affecting the malignant phenotype of glioma cells. However, there was little evidence to suggest that MYC was associated with chemosensitivity of glioma cells. To clarify whether the effects of LINC00470 and miR‐134 on glioma TMZ chemosensitivity were mediated by MYC, we first performed bioinformatics analysis using the TCGA database and found a significant positive correlation between MYC expression and multidrug resistance gene ABCC1 expression in 541 GBM samples (Figure 7A). Similar results were also found in the Oncomine™ gene expression array data sets (https://www.oncomine.org/ resource/main.html) (Figure S2), suggesting that MYC may regulate the expression of ABCC1 and affect the chemosensitivity of gliomas. To verify our hypothesis, we analysed the expression of ABCC1 in MYC‐overexpressing cells. As shown in Figure 7B‐C, overexpression of MYC increased the expression of ABCC1 at both the mRNA and protein levels; on the contrary, overexpression of miR‐134 reduced the expression of ABCC1. When we co‐transfected miR‐134 mimics and MYC plasmid into glioma cells, the down‐regulation of ABCC1 induced by miR‐134 was rescued (Figure 7B‐C), which indicated that miR‐134 regulated the expression of ABCC1 through MYC. Furthermore, we found that ABCC1 was up‐regulated in LINC00470‐overexpressing cells and that this up‐regulation was counteracted when we transfected miR‐134 mimics into LINC00470‐overexpressing cells (Figure 7D‐E). These results indicated that LINC00470 up‐regulated MYC by sponging miR‐134 to regulate the expression of ABCC1, thereby affecting glioma cell TMZ chemosensitivity.

## 4 DISCUSSION

Recently, with more and more evidence showing that lncRNAs function as oncogenes or tumour suppressors, the study of lncRNAs has become increasingly important.^7^ Although the roles of a few lncRNAs in gliomas have been depicted, little is known about LNC00470. Our previous studies have demonstrated that elevated expression levels of LINC00470 were associated with poor prognosis in glioma patients and promoted the glioma growth in an intracranial transplantation mouse model by activating the AKT signalling pathway.^10^ In the present study, we further confirmed that LINC00470 was highly expressed in gliomas and promoted glioma cell proliferation and invasion, and attenuated TMZ chemosensitivity. Our study clarified the new mechanism by which LINC00470 acts as a ceRNA to sponge miR‐134, thereby regulating downstream molecules such as MYC and ABCC1 to maintain the malignant phenotype of glioma.

Given that previous studies report that lncRNAs competitively bind to miRNA response elements in cells as miRNA sponges to regulate mRNA expression,^21^ we speculated that LINC00470 may have a similar mechanism. Using bioinformatics methods, we predicted the binding site of LNC00470 and miR‐134. As expected, we confirmed the direct binding and regulatory relationship between LINC00470 and miR‐134 using a luciferase reporter assay. Unlike LINC00470, miR‐134 is a microRNA that has attracted considerable attention recently; it is considered to be a suppressor in a variety of tumours. For example, Liu et al found that the expression of miR‐134 was significantly down‐regulated in renal cell carcinoma cells and tissues and that the recovery of miR‐134 expression suppressed cell proliferation by silencing the G0/G1 phase.^22^ Overexpression of miR‐134 inhibited the invasion and metastasis of endometrial carcinoma cells.^23^ In non–small‐cell lung cancer, miR‐134 inhibited the expression of cyclin D1/2 and cyclin‐dependent kinase 4 by up‐regulating the expression of P21, thereby inhibiting the proliferation of tumour cells.^24^ In another study, overexpression of miR‐134 suppressed the proliferation of lung cancer cells by down‐regulating the expression of EGFR.^25^ The role of miR‐134 in gliomas has also been identified; it is reported that miR‐134 inhibited the proliferation and invasion of glioma cells by targeting KRAS and activating the ERK pathway.^16^ In a study of GBM, overexpression of miR‐134 significantly suppressed the invasion and metastasis of U87 cells by targeting the *NANOG* gene.^26^ Zhang et al reported that the overexpression of miR‐134 significantly inhibited cell proliferation and xenograft tumour development; furthermore, they suggested a mechanism whereby miR‐134 plays an antitumour role by down‐regulating the expression of KRAS and STAT5B.^14^ These studies are consistent with our results; nevertheless, the relationship between miR‐134 and the TMZ sensitivity of glioma cells has, to the best of our knowledge, never been revealed prior to our study. In addition, we were also the first to identify the direct targeting relationship between miR‐134 and LINC00470. Meanwhile, we found that miR‐134 rescued LINC00470‐induced cell proliferation, invasion and reduction of TMZ chemosensitivity, which indicated that the effect of LINC00470 on glioma was mediated by miR‐134.

In order to explore the downstream effectors of LINC00470 and miR‐134 in glioma cells, we predicted that MYC is the direct target of miR‐134 through bioinformatics analysis. MYC is a member of the helix‐ring‐helix superfamily, which functions as a transcription factor and is highly expressed in various human tumours including gliomas.^27^, ^28^ Importantly, downstream genes regulated by MYC are involved in very important cell functions including cell proliferation, apoptosis, metabolism, protein synthesis, DNA repair, maintenance of cell self‐renewal and tumorigenesis.^29^, ^30^, ^31^ In addition, *MYC* is one of the most frequently deregulated oncogenes currently known, and its forced expression promotes tumorigenesis by providing sufficient energy and metabolic substrates for uncontrolled cell proliferation.^30^, ^32^, ^33^ Previous studies have provided sufficient evidence that MYC is up‐regulated in gliomas and its forced down‐regulation has been shown to promote cell apoptosis and reduce the formation of xenograft tumours in vivo.^34^, ^35^ Our study further confirmed the up‐regulation of MYC at both mRNA and protein levels in gliomas. Some studies have shown that MYC can be regulated by miRNAs at the post‐transcriptional level.^36^ Here, we identified that MYC was a novel target of miR‐134 by dual‐luciferase assay, by which it was regulated. Furthermore, we determined that LINC00470 indirectly regulates the expression of MYC by sponging miR‐134, thus regulating the malignant phenotype and TMZ sensitivity of glioma cells.

Although TMZ is the preferred chemotherapeutic drug for the treatment of GBM, chemotherapy failure is common due to both inherent resistance and acquired resistance.^37^ Therefore, it is meaningful to study the mechanism of TMZ chemosensitivity in glioma cells. MDR is the main cause of chemotherapy resistance, usually mediated by efflux pumps.^5^ Drug efflux induced by efflux pumps occurs based on the ATP‐binding cassette (ABC) superfamily, the mechanism of which is that ABC transporters expressed on the plasma membrane and cell capsule extrude toxins and other foreign substances from cells. At present, 13 of the 48 known ABC transporters have been confirmed to contribute to MDR, including ABCC1.^38^ It is reported that the high expression levels of ABCC1 in various tumours such as lung cancer, oesophageal cancer and colorectal cancer lead to resistance to a variety of anticancer drugs.^39^ Above all, increasing evidence shows that the overexpression of ABCC1 in glioma cells enhances drug resistance.^40^, ^41^ As mentioned previously, *MYC* is an oncogenic gene in gliomas. Although studies have shown that the down‐regulation of MYC is associated with a better prognosis in recurrent GBM patients after TMZ treatment,^42^ the mechanism by which MYC affects TMZ sensitivity remains unclear. Our study revealed a co‐expression relationship between MYC and ABCC1 and that the overexpression of MYC up‐regulated ABCC1, indicating that MYC may affect the drug resistance of glioma cells by regulating ABCC1. Furthermore, we found that LINC00470 up‐regulates the expression of ABCC1, in contrast to miR‐134. Through the rescue experiments, we found that the regulation of ABCC1 expression by LINC00470 was mediated by miR‐134, while the regulation of ABCC1 expression by miR‐134 was mediated by MYC. Based on the above results, we found that LINC00470 regulated MYC by sponging miR‐134 to regulate ABCC1, thus affecting the TMZ sensitivity of glioma cells. That is, LINC00470, miR‐134, MYC and ABCC1 can form a new regulatory axis in gliomas.

Although we identified the role of LINC00470 and miR‐134 in glioma and established the LINC00470/miR‐134/MYC/ABCC1 axis, certain limitations out of our study remain and should be improved upon in the future. For example, in vivo experiments are essential to study the effects of LINC00470 and miR‐134 on TMZ sensitivity. In addition, the knockdown of LINC00470 and miR‐134 would be informative in studying their functions and mechanisms. More biological and clinical studies are necessary to evaluate the utility of targeting the LINC00470/miR‐134/MYC/ABCC1 axis for the treatment of gliomas.

In summary, LINC00470 up‐regulated the expression of MYC by sponging miR‐134 to exert a tumour‐promoting effect, while miR‐134 affected the expression of ABCC1 by targeting MYC. Thus, we identified the LINC00470/miR‐134/MYC/ABCC1 axis and illuminated its biological role and mechanism in glioma. This axis may constitute potential therapeutic targets for gliomas.

## CONFLICT OF INTEREST

The authors declare no conflict of interest.

## AUTHOR CONTRIBUTIONS

**Changwu Wu:** Conceptualization (equal); Data curation (lead); Formal analysis (lead); Writing‐original draft (lead). **Jun Su:** Conceptualization (equal); Data curation (equal); Writing‐original draft (supporting). **Wenyong Long:** Data curation (equal); Writing‐original draft (supporting). **Chaoying Qin:** Data curation (supporting); Formal analysis (supporting). **Xiangyu Wang:** Data curation (supporting); Investigation (supporting). **Kai Xiao:** Data curation (supporting); Formal analysis (supporting). **Yang Li:** Formal analysis (equal). **Qun Xiao:** Formal analysis (supporting); Investigation (supporting). **Junquan Wang:** Data curation (supporting); Investigation (supporting). **Yimin Pan:** Formal analysis (supporting); Investigation (supporting). **Qing Liu:** Conceptualization (equal); Funding acquisition (lead).

## Supporting information

Fig S1

Fig S2

Table S1

## Data Availability

The data sets used and analysed during the current study are available from the corresponding author on reasonable request.
