# Supplementary figures and images for: LINC00470 promotes tumour proliferation and invasion, and attenuates chemosensitivity through the LINC00470/miR‐134/Myc/ABCC1 axis in glioma
Source: J Cell Mol Med. 2020 Sep 11;24(20):12094–106. doi: 10.1111/jcmm.15846 (PMC7579701; doi:10.1111/jcmm.15846)

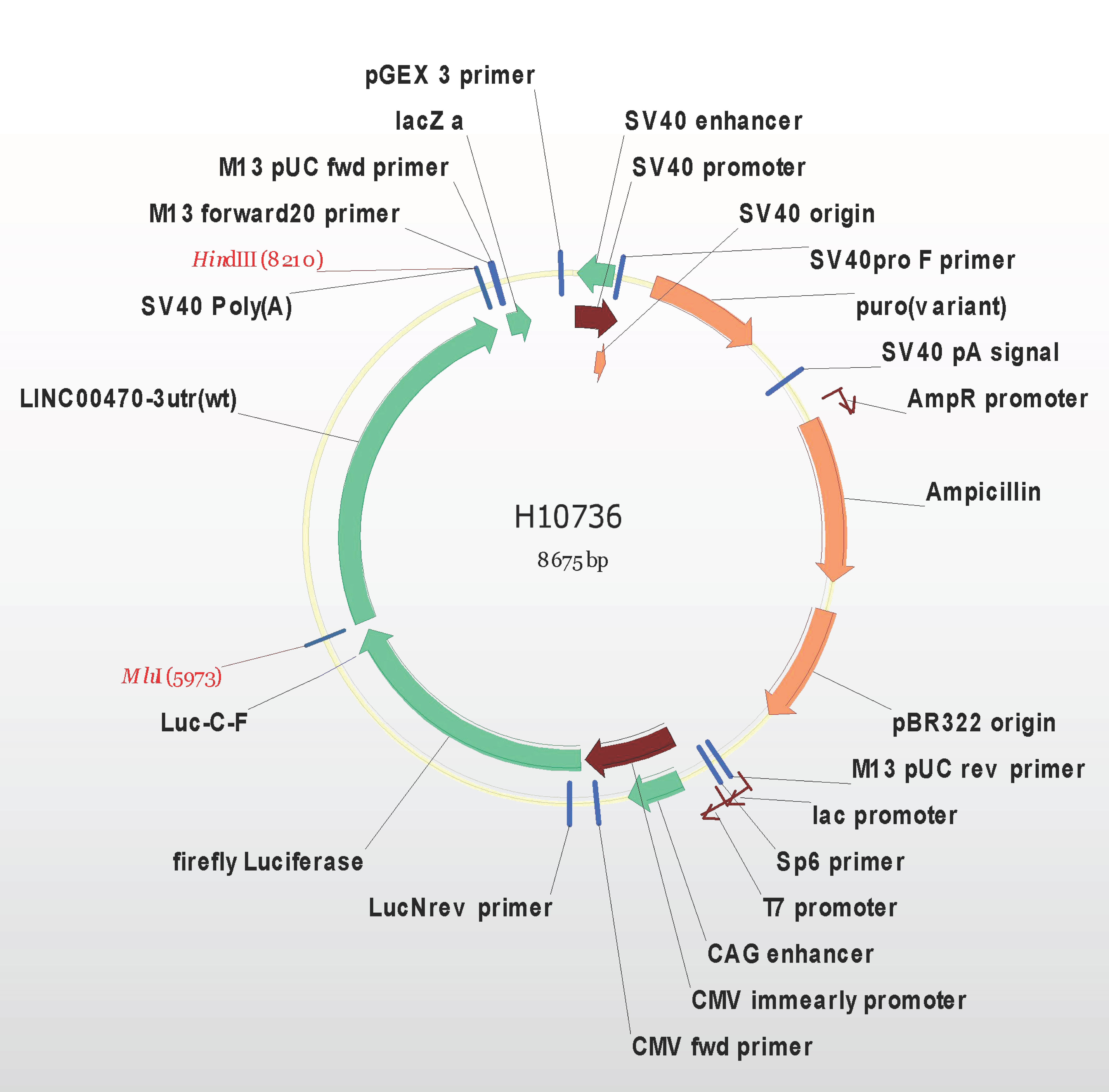

Supplement: Supplementary file 1 — Fig S1 [file JCMM-24-12094-s001.png]

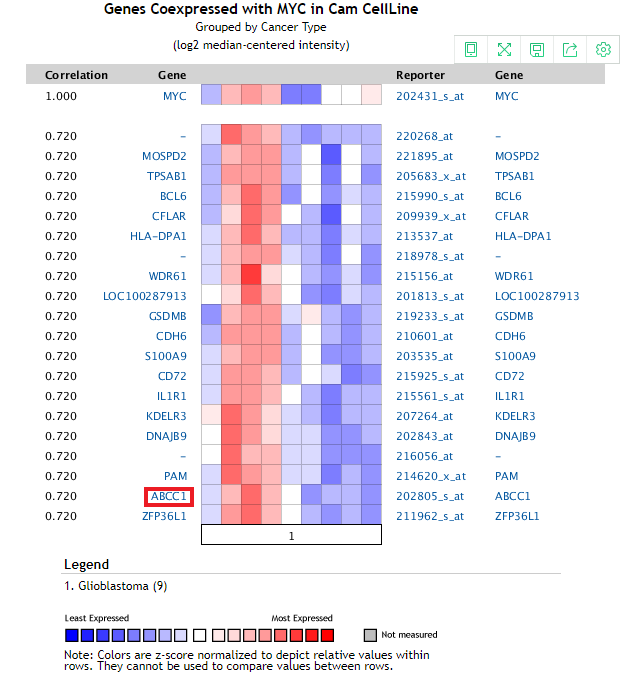

Supplement: Supplementary file 2 — Fig S2 [file JCMM-24-12094-s002.png]
