# Supplementary material for: LINC00470 promotes tumour proliferation and invasion, and attenuates chemosensitivity through the LINC00470/miR‐134/Myc/ABCC1 axis in glioma
Source: J Cell Mol Med. 2020 Sep 11;24(20):12094–106. doi: 10.1111/jcmm.15846 (PMC7579701; doi:10.1111/jcmm.15846)
Supplement: Supplementary file 3 — Table S1 [file JCMM-24-12094-s003.docx]

**Table S1. Clinical parameters of thirty-two glioma** **patients from the Department of Neurosurgery, Xiangya Hospital, Hunan, China**

| Variable |  | Number of  patients | | Percentage  of patients | |
| --- | --- | --- | --- | --- | --- |
| Gender | Male | | 18 | | 56.3% |
|  | Female | | 14 | | 43.7% |
| Age(years) | < 50 | | 12 | | 37.5% |
|  | ≥ 50 | | 20 | | 62.5% |
| WHO Grade | II | | 8 | | 25.0% |
|  | III | | 8 | | 25.0% |
|  | IV | | 16 | | 50.0% |
